# Prognostic Significance of Esophagogastric Junction Invasion in Patients with Adenocarcinoma of the Cardia or Subcardia

**DOI:** 10.3390/cancers15061656

**Published:** 2023-03-08

**Authors:** Sung Eun Oh, Sujin Park, Soomin Ahn, Ji Yeong An, Jun Ho Lee, Tae Sung Sohn, Jae Moon Bae, Min-Gew Choi

**Affiliations:** 1 Department of Surgery, Samsung Medical Center, Sungkyunkwan University School of Medicine, Seoul 06351, Republic of Korea; 2 Department of Pathology, Samsung Medical Center, Sungkyunkwan University School of Medicine, Seoul 06351, Republic of Korea

**Keywords:** cardia and subcardia cancer, Siewert classification, prognosis, TNM staging system, overall survival, esophagogastric junction

## Abstract

**Simple Summary:**

We compared the prognosis of patients with cardia or subcardia cancer using a gastric cancer staging system and identified a significant difference in overall survival according to invasion of the esophagogastric junction. In addition, esophagogastric junction invasion was a significant independent factor reflecting a poor prognosis. Esophagogastric junction invasion should be considered for staging, and additional research is needed to apply it to gastric and esophageal cancer classification.

**Abstract:**

Background: There has been no comparison of the prognoses of Korean patients who underwent curative surgery for cancer located at the cardia or subcardia of the stomach. We performed this comparison and further investigated the prognostic significance of esophagogastric junction (EGJ) invasion in patients. Methods: The medical records of patients (*n* = 511) who were diagnosed with cardia or subcardia cancer and underwent surgery between January 2010 and May 2019 were retrospectively reviewed. Patients were further categorized into four groups for analysis: subcardia gastric cancer (sGC; subcardia cancer without EGJ invasion; *n* = 97), AEG (adenocarcinoma of the esophagogastric junction) type III (subcardia cancer with EGJ invasion, *n* = 54), AEG type II without EGJ invasion (*n* = 158), and AEG type II with EGJ invasion (*n* = 202). We compared the overall survival of the four groups using a gastric cancer staging system and evaluated the prognostic significance of EGJ invasion with multivariate analysis. Results: The median follow-up of patients was 46.0 months (range: 0–124 months). There was significant difference in overall survival curves among the four groups (*p* < 0.001). Subgroup analysis showed a significant difference in overall survival between the groups with and without EGJ invasion (*p* < 0.001). Cancers with EGJ invasion were more frequently in the cardia (*p* < 0.001), had a larger size (*p* < 0.001), and showed a more advanced pathologic stage (stages II and III; 67.6% versus 33.7%, *p* < 0.001) than those without EGJ invasion. EGJ invasion and the pathologic stage were significant independent prognostic factors of overall survival in cardia and subcardia cancer patients (hazard ratio 2.24, 95% confidence interval 1.32–3.81, *p* = 0.003). Conclusion: The overall survival between patients with cardia or subcardia cancer was significantly different according to EGJ invasion. EGJ invasion was an independent prognostic factor and should be considered for staging. Additional research is needed to apply this feature to gastric and esophageal cancer classification.

## 1. Introduction

In recent decades, the incidence of adenocarcinoma of the esophagogastric junction (AEG) has increased significantly in developed Western countries [1,2] and is also increasing in some Asian countries [3,4]. Considering future trends in various risk factors (such as gastroesophageal reflux disease, obesity, H. pylori infection, Barrett’s esophagus) for AEG in Asian countries, the incidence of AEG is likely to gradually increase but not exceed that of AEG in Western countries [4]. These cancers are categorized with the Siewert classification system [5]: type I tumors are those located in the distal esophagus within 1 to 5 cm proximal to the anatomical cardia (above 1~5 cm); type III tumors are located within 2 to 5 cm distal to the anatomical cardia (subcardia, below 2~5 cm) and invade the esophagogastric junction (EGJ); and type II, which is true junctional or cardia cancer, is located within the anatomical cardia (1 cm proximal to 2 cm distal, 1 cm above to 2 cm below).

There have been changes in the staging of cardia and subcardia cancers. In the 7th edition of the American Joint Committee on Cancer (AJCC) [6], AEG type II and type III cancers are staged similarly to adenocarcinoma of the esophagus. In the 8^th^ edition, type II cancers with EGJ invasion are staged using the esophageal cancer staging system, while type II cancers without EGJ invasion and type III cancers are staged using the gastric cancer staging system [7].

Currently, there are discrepancies between the staging and surgical treatment of AEG type II cancers. In Korea, patients with resectable AEG type II or III cancer are recommended to undergo total gastrectomy with D1+ or D2 lymph node dissection [8]. After the operation, gastrointestinal surgeons and pathologists in our institution use the gastric cancer staging system, not the esophageal staging system, for tumors finally diagnosed as type II cancer regardless of EGJ invasion.

AEG types I and III are treated as esophageal cancer and gastric cancer, respectively [9]. However, no consensus has been reached regarding the staging of type II cancers. A previous study indicated that the gastric cancer staging system is superior to the esophageal staging system in the prognosis of type II patients [10]. Another report found that neither system could reflect a prognosis of type II or III because of the different biological properties from pure gastric and pure esophageal cancers [11].

To the best of our knowledge, no recent report has compared the prognosis of patients who underwent curative surgery for AEG type II or type III cancer and whose tumors were staged using the 8^th^ AJCC gastric cancer staging system regardless of EGJ invasion. In this study, we compared the overall survival of patients with cardia or subcardia cancer using the gastric cancer staging system. The cancers were further categorized according to the status of EGJ invasion, and the survival of AEG subgroups was compared. In addition, patients with subcardia gastric cancer were included in this study to compare survival between AEG subgroups. The prognostic significance of EGJ invasion in these cancers was also investigated.

## 2. Materials and Methods

Medical records with pathological reports of patients who were diagnosed with malignancy at the cardia or subcardia of the stomach (*n* = 698) were retrospectively reviewed. The patients underwent surgical treatment between January 2010 and May 2019 at Samsung Medical Center in Seoul, Korea. We excluded patients diagnosed with AEG type I (epicenter located 1 cm~5 cm above the EGJ) invading the EGJ or cardia (*n* = 65), other cancers (*n* = 26), or double primary cancer (*n* = 10). Patients who underwent preoperative chemotherapy/radiotherapy (*n* = 43) or non-curative resection (*n* = 31) and those who were finally diagnosed with stage IV (*n* = 8) were also excluded. In addition, four patients who could not be accurately classified according to the Siewert classification because of insufficient medical records and whose EGJ involvement was uncertain at the time of surgery were excluded from the analysis. A total of 511 patients were included in the final patient group.

**Figure 1:**
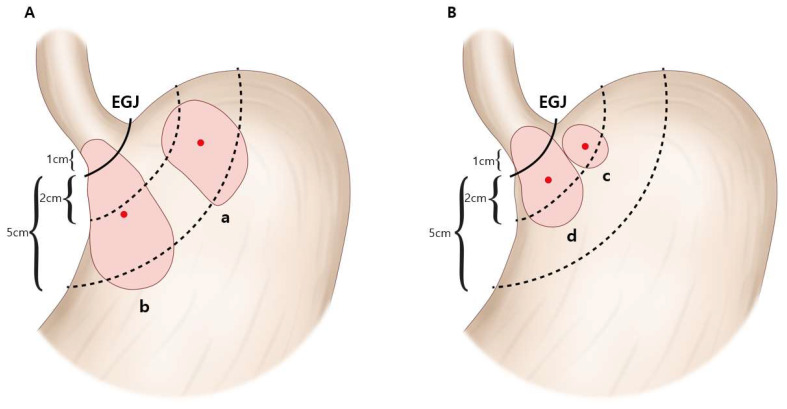
Adenocarcinomas of the cardia and the subcardia. Cardia and subcardia cancers were classified into four categories according to the location of the epicenter (red dots) and invasion of the esophagogastric junction (EGJ). (**A**) When the epicenter is located at the subcardia, the tumor is defined as either subcardia gastric cancer (without EGJ invasion) (a) or AEG (adenocarcinoma of the esophagogastric junction) type III (with EGJ invasion) (b). (**B**) When the epicenter is located at the cardia, the tumor is defined as AEG type II without EGJ invasion (c) or with EGJ invasion (d). AEG: adenocarcinoma of the esophagogastric junction; EGJ: esophagogastric junction.

The 511 patients were further categorized into groups based on the Siewert classification [5]. According to this system, the type of AEG is defined by the location of the epicenter. The epicenter of an AEG tumor can be located at either the cardia (1 cm above to 2 cm below the EGJ) or at the subcardia (2~5 cm below the EGJ). AEG type II is defined when the epicenter of the tumor is located at the cardia. AEG type III is defined when the epicenter of the tumor is located at the subcardia and simultaneously invades the EGJ. When the epicenter of adenocarcinoma is located at the subcardia but does not invade the EGJ, it was classified as subcardia gastric cancer (sGC), which is an upper-third gastric cancer and does not meet the criteria of AEG classification suggested by Siewert et al. Finally, the categorized groups are as follows (Figure 1): (a) sGC (*n* = 97); (b) AEG type III (*n* = 54); (c) AEG type II without EGJ invasion (*n* = 158); and (d) AEG type II with EGJ invasion (*n* = 202). The Siewert type was determined from endoscopic images, intraoperative surgical records (by surgeons), and postoperative pathological specimens (by pathologists). Proximal or total gastrectomy (D1+/D2 lymphadenectomy according to the Korean Gastric Cancer Treatment guideline [8]) was performed on all patients. The median follow-up period was 46.0 months (range: 0–124 months).

The clinicopathologic characteristics (age, sex, postoperative chemotherapy, EGJ invasion, histologic type, Lauren type, tumor size, depth of invasion, lymph node metastasis, pathologic stage, lymphatic invasion, venous invasion, and perineural invasion) were reviewed. The histologic type was dichotomized as differentiated or undifferentiated. The differentiated group was categorized into well- and moderately differentiated adenocarcinomas. The undifferentiated group included poorly differentiated, signet ring cell, and mucinous adenocarcinoma. The pathologic stage was classified according to the 8th edition of the AJCC classification [7]. However, we used the stomach cancer staging system for patients with AEG type II cancers with EGJ invasion for comparison with those of the other three groups. Survival data were obtained from updated medical records and the National Statistical Office in Korea. This study was approved by the Institutional Review Board of Samsung Medical Center (SMC 2021-03-028).

### Statistical Analysis

We used the Chi-square test for categorical variables and Student’s t-test or the Kruskal–Wallis test for continuous clinicopathologic variables. The five-year overall survival was calculated using the Kaplan–Meier method with the log-rank test. Variables with *p* < 0.05 in univariate analysis were selected for inclusion in multivariate analysis using the Cox proportional hazards model with backward logistic regression to identify independent prognostic factors. *p* < 0.05 was considered to indicate statistical significance. The survival curves were statistically different between two groups when *p* < 0.05/4 = 0.0125 in subgroup analysis. Statistical analysis was performed using the Statistical Package for the Social Sciences version 27.0 for Windows (IBM Corporation, Armonk, NY, USA).

## 3. Results

**Figure 2:**
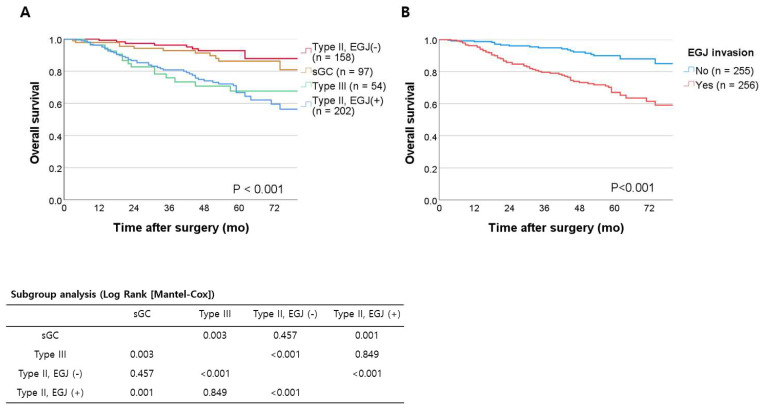
(**A**) Overall survival of cardia or subcardia cancer patients. (**B**) Overall survival of cardia or subcardia cancer patients categorized by EGJ invasion. EGJ: esophagogastric junction; sGC: subcardia gastric cancer without esophagogastric junction invasion.

We compared the clinicopathologic characteristics between the groups with sGC, type III, type II without EGJ invasion, and type II with EGJ invasion, as shown in Table 1. There was no difference in age or sex ratio between groups. Undifferentiated histology (68.5%) and a diffuse Lauren type (55.0%) were most frequent in the type III group, while differentiated histology (56.9%) and an intestinal Lauren type (70.0%) were most frequent in the type II with EGJ invasion group (histologic type *p* = 0.004; Lauren type *p* = 0.036). The mean tumor size was largest in the type III group (*p* < 0.001). Approximately 94.4% of patients with type III and 60.4% of patients with type II with EGJ invasion were diagnosed with an advanced pathologic stage (II and III). Lymphatic invasion and perineural invasion were more frequently found in type III and type II with EGJ invasion groups than in the sGC or type II without EGJ invasion group (*p* < 0.001). The overall survival curves of the four patient groups are shown in Figure 2A. There was a significant difference in overall survival among the four groups (*p* < 0.001). The five-year overall survival of patients was 86.3% in the sGC group, 67.6% in the AEG type III group, 92.8% in the type II without EGJ invasion group, and 66.7% in the type II with EGJ invasion group.

In the subgroup analysis (Figure 2A), there was a significant difference in overall survival between groups with and without EGJ invasion (*p* < 0.0125): sGC versus type III (*p* = 0.003), sGC versus type II with EGJ invasion (*p* = 0.001), type III versus type II without EGJ invasion (*p* < 0.001), and type II with EGJ invasion versus type II without EGJ invasion (*p* < 0.001). No statistical difference was found in overall survival between sGC and type II without EGJ invasion (*p* = 0.457) or between type III and type II with EGJ invasion (*p* = 0.849). Overall survival was significantly different between patients with EGJ invasion and those without (*p* < 0.001, Figure 2B); the five-year overall survival was 90.0% in patients without EGJ invasion and 67.1% in those with EGJ invasion.

The comparison of clinicopathologic characteristics between patients with EGJ invasion and those without EGJ invasion is shown in Table 2. EGJ invasion was more frequent in the cardia group (78.9% versus 62.0%, *p* < 0.001). A larger tumor size was seen in the EGJ invasion group compared with the non-EGJ-invaded group (5.0 ± 2.4 (mean ± SD) versus 3.7 ± 1.8; *p* < 0.001). The frequency of advanced pathologic stages (stages II and III), including the T stage (T3 and T4) and N stage (N1–3), was significantly higher in patients with EGJ invasion than in those without EGJ invasion (all variables, *p* < 0.05).

Univariate and multivariate analyses of overall survival in patients with cardia or subcardia cancer are shown in Table 3. EGJ invasion was a significant factor of overall survival in the univariate analysis (hazard ratio 3.40, 95% confidence interval 2.09–5.55, *p* < 0.001). It also showed significance in the multivariate analysis of overall survival, along with the pathologic stage (hazard ratio 2.24, 95% confidence interval 1.32–3.81, *p* = 0.003, model 1) and TN stage (model 2).

## 4. Discussion

The overall survival was significantly poorer in cardia or subcardia cancer patients with EGJ invasion compared to those without EGJ invasion. Cancers with EGJ invasion were more advanced than those without EGJ invasion. When the patients were stratified according to EGJ invasion, there was a significant difference in overall survival. In addition, EGJ invasion was an independent prognostic factor in patients with cardia or subcardia cancer.

The Siewert classification is used to categorize AEGs according to the anatomical location [5]. In the Korean Gastric Cancer Treatment guidelines, type I cancers are treated as esophageal cancers, and type II/III cancers are treated as gastric cancers [8]. However, there has been a discrepancy between treatment guidelines and previous staging systems. In the 7th edition of the TNM staging system, AEGs were staged as esophageal rather than gastric cancers, regardless of their Siewert classifications [6]. Previous studies have shown that gastric cancer staging is better for classifying patients with type II/III AEGs than is the esophageal cancer staging system [12,13]. However, these results were not fully reflected in the 8th AJCC gastric cancer staging system. In that edition, a tumor with an epicenter located >2 cm distal to the EGJ and invading the EGJ (which is defined as Siewert type III) or a tumor located within 2 cm of the EGJ (Siewert type II) but without the involvement of the EGJ is recommended to be classified as stomach cancer. However, a tumor with an epicenter located within 2 cm of the EGJ and with the involvement of the EGJ is recommended to be classified as esophageal cancer [7].

We used the gastric cancer staging system and found that the AEG type III group showed a poor prognosis similar to that of the AEG type II with EGJ invasion group. Since the AEG type III group and AEG type II with EGJ invasion group showed a different prognosis from gastric cancer located in the subcardia, the application of the esophageal cancer staging system to these groups can be considered. However, further validation of the prognoses of the two groups is needed. In our study, the prognosis differed according to EGJ invasion rather than the location of the epicenter. This implies that the status of EGJ invasion needs to be considered when determining the prognosis of cardia or subcardia cancers, or that a new staging system might be needed in addition to the esophageal and gastric cancer staging systems.

This study revealed that cardia or subcardia cancer patients with EGJ invasion showed significantly worse prognoses compared with those without EGJ invasion, which is similar to the result of a previous study [14]. This result may be because of the distinctive histologic features of EGJ. The precise location of EGJ is the point of the flare of the distal esophagus and the proximal end of the gastric fold [15,16], and there is no serosal layer around the EGJ or cardia of the stomach [17,18], which might be the last barrier against tumor invasion. Furthermore, EGJ invasion in cardia or subcardia cancers might reflect the aggressiveness of the primary tumor. Other possible reasons for the poor outcome in the EGJ invasion group are tumor invasiveness and technical difficulties of surgery [14].

This study has several limitations. This was a retrospective study with information bias. A very small number of patients were diagnosed with AEG type I cancer in our department during the study period; therefore, we could not accurately compare prognoses between type I and type II cancers. Because our institution uses the AJCC 8^th^ edition of the gastric cancer staging system for AEG type II regardless of EGJ invasion, we could not categorize type II cancer patients with the esophageal staging system or directly compare the systems. Further studies should determine whether the esophageal or gastric staging system is more appropriate for not only type II cancer but also for type III cancer, especially for patients with EGJ invasion.

## 5. Conclusions

In conclusion, there was no survival difference between AEG type II with EGJ invasion and type III patients. EGJ invasion in cardia or subcardia cancer patients significantly worsened their prognosis. Whether AEG type III should be reclassified as esophageal cancer like type II with EGJ invasion is an important question. Alternatively, the application of the gastric cancer staging system for type II patients may be possible, but further classification according to EGJ invasion in type II cancer or the application of a new staging system for cardia or subcardia cancer should be considered. The amendment of the next edition is scheduled to be published in 2024 and will be the basis for additional studies.

## Figures and Tables

**Table 1:** Clinicopathologic characteristics of patients diagnosed with cardia or subcardia cancer categorized according to location of the epicenter and invasion of the esophagogastric junction.

Characteristic	sGC	Type III	Type II EGJ (−)	Type II EGJ (+)	*p* Value *
AJCC 7th classification	Gastric	Esophageal	Esophageal	Esophageal	
AJCC 8th classification	Gastric	Gastric	Gastric	Esophageal	
No. of patients	97	54	158	202	
Epicenter	subcardia	subcardia	cardia	cardia	
EGJ invasion	no	yes	no	yes	
Age, years	61.0 ± 10.6	60.1 ± 11.9	60.0 ± 11.0	61.9 ± 10.6	0.354 ^†^
Sex					0.804
Female	20 (20.6)	14 (25.9)	34 (21.5)	40 (19.8)	
Male	77 (79.4)	40 (74.1)	124 (78.5)	162 (80.2)	
Postoperative chemotherapy ^‡^					<0.001
No	60 (64.5)	12 (26.7)	113 (72.4)	94 (50.3)	
Yes	33 (35.5)	33 (73.3)	43 (27.6)	93 (49.7)	
Histologic type					0.004
Differentiation	44 (45.4)	17 (31.5)	71 (44.9)	115 (56.9)	
Undifferentiation	53 (54.6)	37 (68.5)	87 (55.1)	87 (43.1)	
Lauren type ^‡^					0.036
Intestinal	46 (61.3)	18 (45.0)	73 (60.8)	91 (70.0)	
Diffuse	29 (38.7)	22 (55.0)	47 (39.2)	39 (30.0)	
Tumor size, cm	4.5 ± 2.0	7.6 ± 1.6	3.2 ± 1.4	4.3 ± 2.0	<0.001 ^†^
Depth of invasion					<0.001
T1 and T2	61 (62.9)	6 (11.1)	132 (83.5)	107 (53.0)	
T3 and T4	36 (37.1)	48 (88.9)	26 (16.5)	95 (47.0)	
LN metastasis					<0.001
N0	62 (63.9)	12 (22.2)	123 (77.8)	93 (46.0)	
N+	35 (36.1)	42 (77.8)	35 (22.2)	109 (54.0)	
Pathologic stage					<0.001
I	55 (56.7)	3 (5.6)	114 (72.2)	80 (39.6)	
II and III	42 (43.3)	51 (94.4)	44 (27.8)	122 (60.4)	
Lymphatic invasion					<0.001
No	61 (62.9)	19 (35.2)	111 (70.3)	105 (52.0)	
Yes	36 (37.1)	35 (64.8)	47 (29.7)	97 (48.0)	
Venous invasion ^‡^					0.064
No	87 (89.7)	42 (80.8)	147 (93.0)	158 (86.3)	
Yes	10 (10.3)	10 (19.2)	11 (7.0)	25 (13.7)	
Perineural invasion ^‡^					<0.001
No	71 (73.2)	22 (41.5)	128 (81.0)	133 (66.2)	
Yes	26 (26.8)	31 (58.5)	30 (19.0)	68 (33.8)	

Values are represented as mean ± standard deviation or number of patients (percentage). Calculated by * Chi-square test and ^†^ Kruskal–Wallis test. ^‡^ Missing data regarding postoperative chemotherapy (*n* = 30); other types (mixed and indeterminate) or Lauren type (*n* = 146); pathologic reports regarding venous invasion (*n* = 21) or perineural invasion (*n* = 2). AJCC: American Joint Committee on Cancer; EGJ: esophagogastric junction; LN: lymph node; sGC: subcardia gastric cancer without esophagogastric junction invasion.

**Table 2:** Clinicopathologic characteristics of patients diagnosed with cardia or subcardia cancer categorized according to invasion of the esophagogastric junction (EGJ).

Characteristic	EGJ (−) (*n* = 255)	EGJ (+) (*n* = 256)	*p* Value *
Age, years	60.4 ± 10.8	61.5 ± 10.9	0.251 ^†^
Sex			0.982
Male	201 (78.8)	202 (78.9)	
Female	54 (21.2)	54 (21.1)	
Postoperative chemotherapy			<0.001
No	173 (67.8)	106 (41.4)	
Yes	76 (29.8)	126 (49.2)	
Unknown	6 (2.4)	24 (9.4)	
Epicenter			<0.001
Cardia (1 cm above to 2 cm below the EGJ)	158 (62.0)	202 (78.9)	
Subcardia (2~5 cm below the EGJ)	97 (38.0)	54 (21.1)	
Histologic type			0.144
Differentiated	115 (45.1)	132 (51.6)	
Undifferentiated	140 (54.9)	124 (48.4)	
Lauren type			<0.001
Intestinal	119 (46.7)	109 (42.6)	
Diffuse	76 (29.8)	61 (23.8)	
Others	60 (23.5)	55 (21.5)	
Unmentioned	0 (0.0)	31 (12.1)	
Tumor size, cm	3.7 ± 1.8	5.0 ± 2.4	<0.001 ^†^
Depth of invasion			<0.001
T1	137 (53.7)	65 (25.4)	
T2	56 (22.0)	48 (18.8)	
T3	42 (16.5)	93 (36.3)	
T4	20 (7.8)	50 (19.5)	
LN metastasis			<0.001
N0	185 (72.5)	105 (41.0)	
N1	26 (10.2)	48 (18.8)	
N2	31 (12.2)	47 (18.4)	
N3	13 (5.1)	56 (21.9)	
Pathologic stage			<0.001
I	169 (66.3)	83 (32.4)	
II	47 (18.4)	73 (28.5)	
III	39 (15.3)	100 (39.1)	
Lymphatic invasion			<0.001
No	172 (67.5)	124 (48.4)	
Yes	83 (32.5)	132 (51.6)	
Venous invasion ^‡^			0.021
No	234 (91.8)	200 (85.1)	
Yes	21 (8.2)	35 (14.9)	
Perineural invasion ^‡^			<0.001
No	199 (78.0)	155 (61.0)	
Yes	56 (22.0)	99 (39.0)	

Values are presented as mean ± standard deviation or number of patients (percentage). Calculated by * Chi-square test and ^†^ Student’s *t*-test. ^‡^ No pathologic reports regarding venous invasion (*n* = 21) or perineural invasion (*n* = 2). EGJ: esophagogastric junction; LN: lymph node.

**Table 3:** Univariate and multivariate analyses of overall survival in patients diagnosed with cardia or subcardia cancers.

Variable	Univariate Analysis			Multivariate Analysis (Model 1)			Multivariate Analysis (Model 2)		
	HR	95% CI	*p* Value	HR	95% CI	*p* Value	HR	95% CI	*p* Value
Age, years	1.03	1.01–1.05	0.012	1.03	1.01–1.06	0.005	1.04	1.01–1.06	0.002
Sex			0.939						
Male versus Female									
Postoperative chemotherapy			<0.001			0.932			0.744
Yes versus No	2.86	1.80–4.53							
Unknown versus No	4.90	2.38–10.11							
Epicenter			0.566						
Subcardia versus Cardia									
EGJ invasion			<0.001			0.003			0.030
Yes versus No	3.40	2.09–5.55		2.24	1.32–3.81		1.86	1.06–3.24	
Histologic type			0.348						
Differentiated versus Undifferentiated									
Lauren type			<0.001			0.008			0.077
Diffuse versus Intestinal	2.15	1.32–3.52		2.37	1.40–4.00				
Others versus Intestinal	1.00	0.51–1.96		1.18	0.59–2.35				
Unmentioned versus Intestinal	3.43	1.78–6.63		1.98	0.66–5.97				
Tumor size, cm	1.18	1.09–1.28	<0.001			0.719			0.870
Depth of invasion			<0.001						0.023
T2 versus T1	1.67	0.79–3.53					1.56	0.67–3.64	
T3 versus T1	3.73	2.05–6.79					2.27	1.01–5.11	
T4 versus T1	7.05	3.80–13.08					3.99	1.62–9.83	
LN metastasis			<0.001						0.022
N1 versus N0	2.13	1.13–4.04					0.81	0.37–1.74	
N2 versus N0	2.01	1.07–3.78					0.63	0.27–1.47	
N3 versus N0	6.32	3.82–10.47					1.84	0.90–3.77	
Pathologic stage			<0.001			0.001			
II versus I	3.24	1.76–5.95		1.72	0.86–3.42				
III versus I	5.68	3.33–9.68		3.09	1.68–5.69				
Lymphatic invasion			<0.001			0.718			0.853
Yes versus No	2.28	1.50–3.48							
Venous invasion			<0.001			0.001			0.003
Yes versus No	3.30	1.96–5.56		2.51	1.43–4.43		2.33	1.33–4.09	
Perineural invasion			<0.001			0.420			0.990
Yes versus No	3.04	2.00–4.63							

Variables with *p* < 0.05 in the univariate analysis were selected for inclusion in the multivariate analysis using the Cox proportional hazards model with backward logistic regression. Variates in Model 1 analysis: variables with *p* < 0.05 in the univariate analysis, except T stage (depth of invasion) and N stage (LN metastasis). Variates in Model 2 analysis: variables with *p* < 0.05 in the univariate analysis, except pathologic stage. CI: confidence interval; EGJ: esophagogastric junction; HR: hazard ratio; LN: lymph node.

## Data Availability

The datasets used and/or analyzed during the current study are available from the corresponding author on reasonable request.
